# The modified single patch technique

**DOI:** 10.4103/0974-2069.52809

**Published:** 2009

**Authors:** Ralph S Mosca, Jan M Quaegebeur

**Affiliations:** Department of Surgery, Division of Cardiothoracic Surgery, Columbia University College of Physicians and Surgeons, New York, USA

Repair of atrioventricular septal defects has undergone a number of modifications over the last 40 years. Lillehei and colleagues are credited with the first successful repair of a complete atrioventricular septal defect (CAVSD) in 1954.[1] The late 1950s and early 1960s saw a number of small series of repairs of CAVSDs, most utilizing the “two-patch technique”. In 1962, Maloney *et al*. reported the “one-patch technique,” which in their hands led to improved survival.[2] Details of the anatomy of CAVSD, including the morphology of the atrioventricular valves, ventricular and atrial septal defects, the unique structure of the left ventricular outflow tract (LVOT), and the location of the conduction system, led to an improved understanding, which translated into better patient outcomes.

The purported benefits and pitfalls of both single- and two-patch techniques have been touted by multiple authors. Subsequently, Wilcox and Anderson described the repair of a ventricular component of the CAVSD by directly approximating the left atrioventricular (AV) valve component to the scooped out intraventricular septum.[3] Nunn and others continued the experience with this approach, with good short-term results.[4] Dr. Backer and colleagues now have a series of 26 patients with short-term follow up. Their approach is now referred to as the “modified single patch technique”.[5]

Repair of CAVSD at the Children's Hospital of New York (CHONY) is usually undertaken with a two-patch technique, essentially as outlined by Dr. Backer in one of the groups' earlier publications.[6] Bicaval venous cannulation with moderate hypothermia (28-32°C) and aortic cross clamping is utilized. As opposed to what Dr. Backer has described, we find it helpful to put two separate sutures, one in the left superior leaflet (LSL) and one “kissing it” in the left inferior leaflet (LIL), to line up the base of the cleft. This allows the sutures to be separated, retracting the left atrioventricular valve (LAVV) components and greatly improving visualization of the ventricular septal defect (VSD) margins. This is particularly important when the VSD extends high up into the outlet septum. Furthermore, the LSL is never intentionally divided.

The VSD component of the defect is then closed, followed by suturing of the top of the patch to the crest of the LAVV component and a pericardial patch. The “cleft” in the LAVV is closed with interrupted horizontal mattress sutures, without pledgets. Of note, the VSD patch is undersized slightly in both width and height, to avoid separating the LAVV components or elevating the valve leaflets above the natural height of the annulus of the common AV valve, both of which can promote regurgitation. This undersizing of the patch creates a type of “septal annuloplasty”. Occasionally, “Wooler type” commissuroplasty stitches are placed between the LSL and the left lateral leaflet or between the left inferior bridging leaflet and the left lateral leaflet. Closure of the cleft is done routinely unless the LAVV is inherently small or a parachute or double orifice type anatomy is encountered. The coronary sinus (CS) is left to drain into the right atrium, unless the “coronary sinus septum” that is between the mouth of the CS and the primum ASD is significantly deficient.

At CHONY we now have a limited experience (approximately 10 patients) with the modified single-patch technique also showing good results. Due to surgeon preference, the modified single-patch technique has been used in those instances in which the VSD component has been nonrestrictive, but the height of the defect has been limited. In other words, the degree of scooping out of the ventricular septum has been mild. Dr. Backer notes that the VSDs in his patients are about 9 mm, however, it is not clear what dimension this is describing. As one would expect, we have also noted a decrease in the aortic cross clamp and total cardiopulmonary bypass times when using the modified single-patch technique. Early results reveal no mortalities, essentially minimal LAAV regurgitation, no instances of complete heart block or subaortic stenosis, and mild-to-moderate right atrioventricular valve (RAVV) regurgitation.

When examining the two techniques, the major components of repair and possible complications need to be assessed and compared.

## LEFT ATRIVENTRICULAR VALVE REGURGITATION

Key points for achieving competency of the LAVV, by whichever technique is employed, include, accurately evaluating and aligning the limits of the LSL and LIL bridging leaflets, suturing these leaflets to one another as well as to the appropriate point along the scooped out portion of the interventricular septum, and avoiding prolapse of the valve.

A benefit of the use of a VSD patch is that the LAVV can be floated and sutured directly to the point where it abuts the VSD patch. Care must be taken to avoid “oversizing” the patch so as not to stretch the annulus open, distort the valve chordae or cause the LAVV to rise above the level of the annulus. When no VSD patch is used, one must rely upon the “minds eye” to identify the correct point on the septum (cephalad to caudal dimension).

The current reported incidence of reoperation for “LAAV” insufficiency after the modified single-patch technique, in this series, is low. This may be a significant improvement over previously reported methods or a result of limited follow-up.

## ASD CLOSURE

Closure of the atrial component of the defect is similar regardless of the method used. The decision as to whether or not to leave the CS draining into the right atrium or incising it and allowing the coronary venous flow to drain to the left atrium should be individualized. The incidence of heart block has been quite low even when sewing in the vicinity of the conduction pathway, as it travels toward the ventricular septum. Cases in which there is very deficient tissue between the coronary sinus and the primum ASD may leave the conduction system more vulnerable and may be best treated by incorporating the CS into the left atrium. As a caveat, some surgeons have been concerned that the coronary sinus connections to the atrial septum and to the LAVV annulus contribute to the support of the LAVV, and incising it may allow the annulus to stretch open and increase the insufficiency.

## VSD CLOSURE

When using the “two-patch technique” the VSD is closed by filling the defect with a prosthetic patch. This is often easier to do when dealing with a Rastelli C type defect (typically consists of a large “scooped out” VSD with few chordal attachments to the septum) rather than a Rastelli type A defect. The usual VSD in CAVSD is larger in its midportion and in the subaortic area than it is in the more inferior area of the coronary sinus. The patch must be trimmed accurately so as to avoid distortion of the chords and LAVV. In general, it is better to undersize rather than oversize this patch.

Closure of the VSD by the “modified single-patch” technique is accomplished by bringing the septal components of the LSL and LIL down to the intraventricular septum.

In instances with multiple chords crossing the defect, this method may be easier than placing a patch around these chordae.

## LEFT VENTRICULAR OUTFLOW TRACT OBSTRUCTION

In CAVSD the LVOT is relatively narrow when compared to that of a normal heart as a result of: (i) anterior displacement of the outflow tract (ii) apical displacement of the AV valve orifices (iii) accentuated area of direct fibrous continuity between the aortic valve and LSL (iv) prominence of the muscle of Moulaert protruding into the LVOT, and (v) tendency for fibromuscular obstruction in the subaortic area due to accessory LAVV tissue and abnormal papillary muscle insertions.

Given the above, one might predict that primary closure of the VSD would lead to left ventricular outflow tract obstruction (LVOTO). Thus far, this appears not to be the case, at least in short-term follow-up. Instances in which the LVOT appears unusually small, prior to the operation, and those in which the VSD extends into the subpulmonary area, that is, Tetralogy of Fallot with CAVSD, may still require a prosthetic patch to minimize postoperative LVOTO. These details will be borne out with further follow up.

## RIGHT ATRIOVENTRICULAR VALVE REGURGITATION

Following repair with the two-patch technique, tricuspid valve competency is accomplished by the septal component of the RAVV abutting the VSD patch-LAVV suture line. The tricuspid valve closes at the same level as the LAVV and if necessary can be sutured to the above.

As mentioned above, the modified single-patch technique draws the LAVV component down to the IV septum. The RAVV portion, however still has its closure point “higher” in the right atrium. This may lead to more significant RAVV regurgitation following this method.

## CONCLUSION

Repair of CAVSD continues to evolve. Better understanding of the anatomy of the atrial and ventricular septal defects, conduction system, atrioventricular valve leaflets, and LVOT have led to improved results over time. Each technique; single-patch technique, two-patch technique, and modified single-patch technique may offer advantages in certain situations. When critically examined, the modified single-patch technique is essentially the same as the two-patch technique, except for the method of VSD closure. At least in the short-intermediate follow-up, the modified single-patch technique performs much better than many predicted, when pertaining to the function of the LAVV and the LVOT. The utility of the modified single-patch technique in the presence of a very large outlet component of the VSD remains unclear. It is likely that in the future it will behoove the surgeon to be experienced with each method and apply each when most appropriate.
